# Physicochemical, nutritional, and sensory analyses of a nitrate-enriched beetroot gel and its effects on plasmatic nitric oxide and blood pressure

**DOI:** 10.3402/fnr.v60.29909

**Published:** 2016-01-19

**Authors:** Davi Vieira Teixeira da Silva, Fabricio de Oliveira Silva, Daniel Perrone, Anna Paola Trindade Rocha Pierucci, Carlos Adam Conte-Junior, Thiago da Silveira Alvares, Eduardo Mere Del Aguila, Vania Margaret Flosi Paschoalin

**Affiliations:** 1 Departamento de Bioqímica, Instituto de Química, Universidade Federal do Rio de Janeiro, Rio de Janeiro, Brazil; 2 Departamento de Nutrição Básica e Experimental, Instituto de Nutrição, Universidade Federal do Rio de Janeiro, Rio de Janeiro, Brazil; 3 Departamento de Tecnologia e Inspeção de Alimentos, Instituto de Tecnologia de Alimentos, Universidade Federal Fluminense, Niterói, Brazil; 4 Instituto de Nutrição, Nucleo de Nutrição Básica e Dietética, Universidade Federal do Rio de Janeiro, Macaé, Brazil

**Keywords:** beetroot gel, plasma nitrite, blood pressure, antioxidant activity, nitrate supplementation

## Abstract

**Background:**

Beetroot (*Beta vulgaris* L.) is a dietary source of natural antioxidants and inorganic nitrate (${\text{NO}}_{\text{3}}^{-}$). It is well known that the content of antioxidant compounds and inorganic nitrate in beetroot can reduce blood pressure (BP) and the risk of adverse cardiovascular effects.

**Objective:**

The aim of the present study was to formulate a beetroot gel to supplement dietary nitrate and antioxidant compounds able to cause beneficial health effects following acute administration.

**Design and subjects:**

A beetroot juice produced from *Beta vulgaris* L., without any chemical additives, was used. The juice was evaluated by physicochemical and microbiological parameters. The sample was tested in five healthy subjects (four males and one female), ingesting 100 g of beetroot gel.

**Results:**

The formulated gel was nitrate enriched and contained carbohydrates, fibers, saponins, and phenolic compounds. The formulated gels possess high total antioxidant activity and showed adequate rheological properties, such as high viscosity and pleasant texture. The consumer acceptance test for flavor, texture, and overall acceptability of beetroot gel flavorized with synthetic orange flavor had a sensory quality score >6.6. The effects of acute inorganic nitrate supplementation on nitric oxide production and BP of five healthy subjects were evaluated. The consumption of beetroot gel increased plasma nitrite threefold after 60 min of ingestion and decreased systolic BP (−6.2 mm Hg), diastolic BP (−5.2 mm Hg), and heart rate (−7 bpm).

The beet (*Beta vulgaris* L.) is a member of the Chenopodiaceae family and its edible part is the root (1). This tuber is a rich source of fiber, vitamins (folic acid, vitamins A, C, B6, niacin, and biotin), and minerals (iron, magnesium, selenium, potassium, calcium, zinc, phosphorus, and sodium) and has a high nutritional value, predominantly due to its carbohydrate content (2). Regular consumption of fruits and vegetables, sources of several bioactive compounds, has beneficial effects on human health by reducing the risk of developing chronic and cardiovascular diseases (3, 4).

The beetroot is a source of bioactive compounds, including phenolic compounds, saponins, and especially betalains, which are responsible for the characteristic color of this tuber (5). Several studies have reported the antioxidant capacity of the betalains and phenolic compounds present in beets, suggesting a protective role of these compounds regarding oxidative processes (6). The beetroot is a nitrate-rich food product that is absorbed in the proximal intestine and is the source of endogenous ${\text{NO}}_{\text{2}}^{-}$ and NO (7, 8). NO and ${\text{NO}}_{\text{2}}^{-}$ are vasoactive agents with the ability to increase vasodilatation, decrease blood pressure (BP), and improve cardiovascular function in both healthy individuals (7, 9) and hypertensive patients (10). The dietary administration of ${\text{NO}}_{\text{3}}^{-}$ caused an acute reduction in systolic and diastolic BP in healthy subjects, after the ingestion of 500 mL of beet juice (11). In addition, beet intake has been associated with improved performance and increased tolerance to fatigue during exercise (12, 13).

Dietary supplementation of nitrate from beet is challenging with regard to the form of beet intake, in order to provide a ready, easy to administer, attractive, nitrate-rich food product with the aim of promoting beneficial effects on the cardiovascular system.

In the present study, a beetroot gel was formulated for the dietary supplementation of nitrate and antioxidant compounds and its effects on plasma nitrite levels and BP of five healthy volunteers following acute administration were investigated. The beetroot gel proximate composition, fiber, flavonoid, and saponin contents were determined, in addition to total antioxidant activity. The rheological properties of the beetroot gel and its overall acceptability were also addressed.

## Methods

### Standards and reagents

Sodium carboxymethyl-cellulose (CMC), ferrous sulfate heptahydrate, benzene-1,4-dicarboxylic acid (terephthalic acid), hydrogen peroxide, Follin–Ciocalteau reagent, gallic acid, 2,2’-azino-bis (2-ethylbenzothiazoline-6-sulfonic acid), diammonium salt (ABTS), 2,4,6-tris(2-pyridyl)-*S*-triazine, potassium persulfate, and (±)-6-hydroxy-2,5,7,8-tetramethylchromane-2-carboxylic acid (Trolox) were purchased from Sigma-Aldrich Chemical Co. (St. Louis, MO, USA). Sodium carbonate, vanillin, and aluminum chloride were purchased from Spectrum Chemical Manufacturing Corp. (Gardena, CA, USA). Saponin standard from soybean was purchased from Wako Pure Chemical Industries (Osaka, Japan). All solvents were HPLC grade from Tedia (Fairfield, OH, USA). HPLC grade water (Milli-Q system, Millipore, Bedford, MA, USA) was used.

### Samples

All the beetroots used in the present study belonged to the Chenopodiaceae family and *Beta vulgaris* L. species, and were purchased at local markets in the city of Rio de Janeiro, Brazil.

### Beetroot gel formulation and juice preparation

Four kilograms of beetroot tubers were divided into two lots of 2 kg each. Two kilograms of the beetroots were thoroughly washed in tap water, sanitized with a chlorine solution, and put into a centrifuge blender (model CE700, Black & Decker^®^). The juice resulted in 91.55±0.75% moisture without any chemical additives. The second 2 kg lot was sliced and frozen at −20°C for 48 h. Subsequently, the beetroots were freeze dried in a Liotop P1040 model (Liobras, São Paulo, Brazil) and then crushed in a portable blender (Blender Pratic^®^ cadence). The resultant powder (16.8 g) was mixed with 2.8 g CMC, 90 mL beetroot juice and 1 mL orange flavor. For analysis, the mixture was dissolved in water at a concentration of 25% (w/v).

### Physicochemical analyses

#### Rheological analyses

The apparent viscosity of the beetroot gel was determined using a controlled stress rate rheometer (model AR-G2, TA Instruments, New Castle, Delaware, USA) with parallel plate geometry (*D*=40 mm) and a 1,000 µm gap. A gradient with increasing shear rates (0–200 s^−1^) and 2 min to decreasing shear rate (200–0 s^−1^) with system temperature set at 25°C was applied. Both the increasing and descending curves generated 30 points of shear rate *versus* shear stress, resulting in a total of 60 points, where the average value of shear stress for each shear rate was taken. The experimental data were evaluated and fitted according to the rheological models of Power Law (Eq. 1)

1 σ=ŋγ

where *σ* is the tension (Pa), *γ* the deformation rate (s^−1^), and *ŋ* the coefficient of viscosity (Pa.s); using the Origin 7.0 software package to obtain the rheological behavior.

### Color measurements

The color intensity was measured with a Minolta type CR-400 colorimeter (Konika Minolta, Tokyo, Japan), previously calibrated with a white porcelain plate. The instrument recorded L* values (lightness-intensity of white color) on a scale ranging from 0 to 100, where 0 is black and 100 is white; a* values (redness-intensity of red color), where a*>0=red and a*<0=green; and b* values (yellowness-intensity of yellow color), where b*>0=yellow and b*< 0=blue.

### Proximate composition

Moisture, ash, total dietary fibers, protein, and lipid content were determined by the official AOAC methods (14). Total carbohydrates were determined by difference. Each sample was analyzed in triplicate. The results were expressed as g 100 g^−1^ of fresh weight (FW).

### Bioactive compound analyses

#### Nitrate content (${\text{NO}}_{\text{3}}^{-}$)

The gel was dissolved in water at a concentration of 25% (w/v) and then centrifuged at 4,500×g for 10 min. The supernatant was filtered throughout a 0.45-µm cellulose membrane filter (MF Millipore^®^) and then diluted at 1:8,000 ratio. The ${\text{NO}}_{\text{3}}^{-}$ analysis was performed as described previously by Li et al. (15), with some adaptations. ${\text{NO}}_{\text{3}}^{-}$ present in the filtered supernatant was enzymatically converted to nitrite (${\text{NO}}_{\text{2}}^{-}$) by nitrate reductase (EC 1.6.6.2, from *Aspergillus* sp.) (Roche Diagnostics, Mannheim, Germany). After converting ${\text{NO}}_{\text{3}}^{-}$ to ${\text{NO}}_{\text{2}}^{-}$, 100 µL of the filtered sample were incubated at 24°C with 10 µL of 316 mM of 2,3-diaminonaphthalene in 0.62 M HCl for 10 min, followed by the addition of 5 µL of 2.8 M NaOH. Samples were immediately analyzed by reverse-phase HPLC (LC-20A Prominence, Shimadzu^®^, Japan). The HPLC device was equipped with a 5-µm C8 Discovery^®^ column (150×4.6 mm, I.D.) guarded by a 5-µm reversed-phase C18 guard column Ascentis^®^ (50×4.6 mm, I.D.) and a fluorescence detector model RF-10AXL (Shimadzu^®^, Japan) monitoring excitation and emission wavelengths at 375 and 415 nm, respectively. The mobile phase (1.3 mL/min) was 15 mM sodium phosphate buffer (pH7.5) and methanol (50:50, v/v) at gradient elution. Triplicate measures were performed and the nitrate contents were expressed as mmol 100 g^−1^.

### TAP analyses

Total antioxidant potential (TAP) analyses of the beetroot gel and juice samples were performed by HPLC (16). TAP was expressed as the percentage difference between the surface area of the chromatogram generated in the Fenton reaction with and without the sample.

### Spectrophotometric assays

Total phenolic compounds (TPC) of the beetroot gel and juice samples were determined using the Folin–Ciocalteau method (17). Results were expressed as mg of gallic acid equivalents per g (mg GAE g^−1^) of FW.

Total flavonoids in the beetroot gel and juice samples were determined using the modified aluminum chloride spectrophotometric assay (18). Results were expressed as mg of quercetin equivalents per g (mg QE g^−1^) of FW.

Total saponins of the beetroot gel and juice samples were determined by the vanillin-sulfuric acid assay (19). Results were expressed as mg of total saponins per g of FW.

The antioxidant capacity (TAC) of the beetroot gel and juice were determined by ferric reducing antioxidant power (FRAP) and Trolox-equivalent antioxidant capacity (TEAC) assays. The FRAP assay was performed according to Benzie and Strain (20) with slight modifications. The TEAC assay was performed according to Re et al. (21) with slight modifications. FRAP and TEAC results were expressed as µmol of Fe^+2^ equivalents per g of FW and in terms of percentage of inhibition of the ABTS radical cation (ABTS^+^) by antioxidants in the sample, respectively. Each sample was independently analyzed by each assay in triplicate.

### Microbiologic analysis

Gel samples (25 g) were weighed into sterile Stomacher^®^ bags by using sterile tools, and after the addition of 225 mL of a sterile saline solution prepared with MilliQ water, were homogenized for 90 s in a Stomacher^®^400 Circulator masticator (Seward Ltd., Worthing, UK). Decimal dilutions of the samples were prepared until 10^−6^ for the microbiological analyses. Total and fecal coliforms were enumerated by the standard methodology of the most probable number (MPN) and *Bacillus cereus* was enumerated in selective MYP medium (Plast Labor^®^, RJ, Brazil), by the standard methodology of colony-forming units (CFU); both methods according to the American Public Health Association (22). *Salmonella* sp. was enumerated using 3M™ Petrifilm™ plates (3M Health Care, Canada) according to the manufacturer's instructions.

### Acceptance test of beetroot gels

Two beetroot gel samples were evaluated for acceptance, differing only by the addition of an orange synthetic flavor (0.9%). The samples were prepared 24 h before the day of analysis and stored at 5°C in 30 mL plastic cups randomly numbered (three digits). Each sample was evaluated by 101 untrained panelists comprising both males and females using a 9-point hedonic scale, where one reflected extreme dislike and nine reflected the highest acceptability. The sensory attributes evaluated were flavor, aroma, texture, and overall acceptability (23). A score of 5 (neither liked nor disliked) was considered as the indifference region of the affective relationship of the panelist to the product, scores from 6 to 9, as the acceptance region and scores from 1 to 4 as the rejection region. At the same time, the buying intention of the samples were also evaluated, using a 5-point hedonic scale, scored from ‘will certainly purchase’ (score 5) to ‘will certainly not purchase’ (score 1) (24). Experiments were carried out in a closed cabin with white illumination and the samples were randomly served accompanied by a sensory evaluation form.

### Nitric oxide production and BP evaluation

Five healthy subjects (four males and one female, age 27.4±1.94 years; BMI 22.8±3 kg/m^2^) were invited to ingest 100 g of beetroot gel. A written informed consent prior to enrollment was given to the volunteers. The volunteers presented blood biochemical indices (glucose, total cholesterol, low density lipoprotein, high density lipoprotein, triglycerides, glutamic oxaloacetic transaminase, glutamic pyruvic transaminase) and urine (urea, creatinine, uric acid) parameters within normal range. The subjects were advised to restrict their diets regarding foods rich in ${\text{NO}}_{\text{2}}^{-}$ and ${\text{NO}}_{\text{3}}^{-}$ for 24 h before the study. A list describing foods and food groups to be avoided or preferred was distributed to them. This dietary orientation was based on a list developed for the estimation of dietary ${\text{NO}}_{\text{2}}^{-}$ and ${\text{NO}}_{\text{3}}^{-}$ (25). The subjects were also instructed to avoid the practice of strenuous exercise, alcohol, and caffeine intake 24 h before the study. Heart rate (HR), systolic (SBP) and diastolic (DBP) BP were measured before and at 30 min intervals post-beetroot gel ingestion during 3 h. BPs were taken with the subject seated, using an automated BP measuring machine (Omron 705CP, Rio de Janeiro, Brazil). Fasting blood samples (5 mL each) were collected into EDTA tubes for plasma nitrite measurements at baseline and every hour during 3 h, and centrifuged immediately at 2,200×g for 10 min at 4°C. The plasma was collected and stored at −80°C until nitrite concentration measurements. All experimental procedures were performed in accordance with the ethical standards of the Declaration of Helsinki and were approved by the Institutional Ethics Committee of the Hospital Universitário Clementino Fraga Filho, Rio de Janeiro (underNo.15510313.5.0000.5257).

Plasma ${\text{NO}}_{\text{2}}^{-}$ was used as a biomarker for nitric oxide availability (26) as previously described by Li et al. (15). The values were expressed as µmol ${\text{NO}}_{\text{2}}^{-}$ L^−1^.

### Statistical analyses

Data from the nitric oxide production, BP, and HR were analyzed using the GraphPadPrism version 5.0 software for Windows (GraphPad Software, San Diego, CA, USA). Comparisons of plasma nitrite and BP between baseline and post-beetroot gel consumption were analyzed using a one-way ANOVA with Dunnett's *post hoc* tests. Data from sensorial evaluation of beetroot gel were analyzed using the one-way ANOVA and Tukey *post hoc* tests. A *p*<0.05 was considered significant for all analyses. All data were expressed as means±SD.

## Results and discussion

### Apparent viscosity

**Fig. 1:**
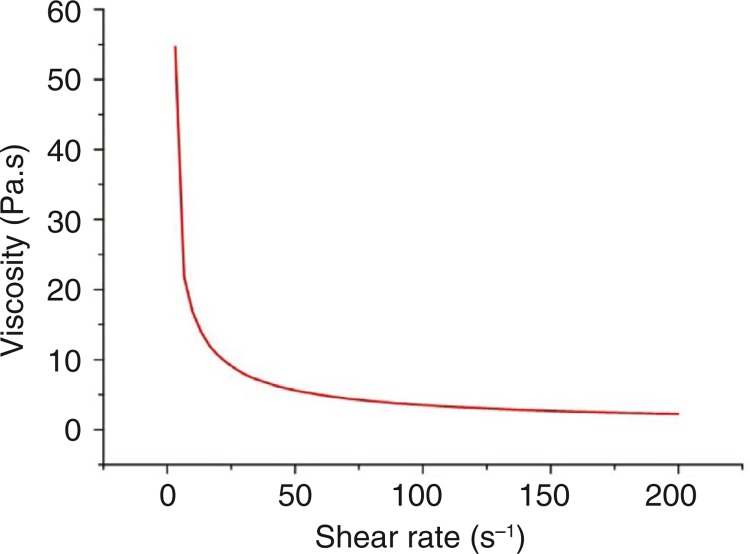
Viscosity analyses of beetroot gel at 25°C.

The beetroot gel exhibited the features of a pseudoplastic fluid, characterized by a decrease in apparent viscosity as a function of increasing shear rate (deformation) (Fig. 1). This behavior can be explained by the increased mobility of the suspended particles in the gel, due to forces generated during the shear. These particles tend to orient themselves in parallel to the direction of the applied force, reducing flow resistance (viscosity). This pseudoplastic behavior may be advantageous for industrial beet gel production and marketing, in terms of handling, packaging, and income, because the product flows smoothly as forces are applied. These rheological characteristics of the gel will result in an adequate flow from the sachet to the mouth during gel ingestion.

According to Rao (27), food rheology has an influence on taste and flavor perception, because of an effect related to the physical properties of the food (i.e. texture, viscosity), which affects the rate and extent of which the stimuli reaches the taste buds.

### Color analyses

Color is one of the most important food attributes and is considered a quality indicator, often determining the food's acceptance (28). The beet gel showed a low lightness (L*) value of 20.12±0.3, according to the CIE system scale ranging from 0 to 100. The gel also showed values of 4.9±0.41 and 1.5±0.13, indicating the presence of the color red (a*>0) and proximity of the color blue (b*<0), respectively. The lightness values (L*), redness (a*), and yellowness (b*) are presented in Table 1.

**Table 1:** Proximate composition of beetroot gel compared to data available in United States Department of Agriculture (USDA)

Nutrient	Beetroot gel (g/100 g)	Beetroot (USDA)
Moisture	76.14±0.48	87.58
Protein	3.02±0.09	1.61
Lipids	0.56±0.01	0.17
Total carbohydrates	13.62±0.31	9.56
Total dietary fiber	4.65±0.28	2.8
Ash	2.01±0.13	–

Data are expressed as means±standard deviation of beetroot gel measurements.

Visually, the gel produced herein had a low brightness (dark) red-violet color, characteristic of raw beets, due to the characteristic color of betanin (that belongs to the betalain class), a water-soluble compound derived from betalamic acid (29).

### Proximate composition

The beetroot gel showed a carbohydrate content of 13.62±0.31 g 100 g^−1^ (Table 2), which is 42.5% higher than the value reported by the USDA of 9.56 g 100 g^−1^ for raw beets. The high carbohydrate content was expected, because the gel was formulated from a mixture of beet juice with dehydrated beets, in which the analyzed nutrients are concentrated due to water removal. The high carbohydrate concentration can be an advantageous feature if the beetroot gel is to be used during physical activity, because carbohydrates can delay fatigue and improve performance (30), as accepted and recommended by the American College of Sports Medicine (31).

**Table 2:** Color properties of the beetroot gel

Color parameters	Value
L*	20.12±0.3
a*	4.9±0.41
b*	1.5±0.13

The gel showed a total fiber content of 4.65±0.28 g 100 g^−1^ (Table 2), higher than raw beets, which showed 2.8 g 100 g^−1^ (2). Insoluble fiber and fiber from cereal and vegetable sources have been inversely associated with the risk of coronary heart disease and cardiovascular disease (32).

### ${\text{NO}}_{\text{3}}^{-}$ content

Inorganic nitrate (${\text{NO}}_{\text{3}}^{-}$) content (Table 2) found in beetroot gel was of 6.3±0.41 mmol 100 g^−1^, significantly higher than the value obtained for beetroot juice processed at the laboratory (1.6±0.1 mmol 100 g^−1^; *d*=1 g/mL). Wruss et al. (33) determined the nitrate concentration of 16 commercial sugar beet juices, which ranged from 0.23 to 6.4 mmol 100 g^−1^. Several factors influence nitrate levels in vegetables and fruits, including season, light intensity and exposure, temperature, growing conditions and the use of fertilizers and storage, which explains the large difference in nitrate levels in beetroot (34).

Clinical studies assessing the effect of the ingestion of nitrate beet juice on cardiovascular parameters, administered beet juice doses, with nitrate concentrations ranging from 5.5 to 6.4 mmol in 250 g with beneficial changes on cardiovascular health (10, 35). The beetroot gel showed higher nitrate concentrations (6.3 mmol in 100 g), allowing the dietary nitrate supplementation can be conducted without discomfort, in the same way it is done with traditional carbohydrate gels, commonly consumed during intense physical exercise. The substitution of beetroot juice with nitrate-enriched gels avoids the intake of large volumes of liquid.

### Antioxidant potential, phenolic compounds, antioxidant activity, flavonoid, and saponin contents of beetroot gel

The TAP, TPC, flavonoid, and saponin content of the beetroot gel were compared to the beetroot juice, because the juice is the most widely used form of this food in bioactivity studies and clinical trials (36).

The gel showed significantly higher TAP values than the juice, 87±0.1 vs. 79.13±0.63% (Table 3), reflecting the different antioxidants presents in beetroot gel able to quench the hydroxyl radical, the most reactive radical in living organisms (37).

**Table 3:** Antioxidant potential, antioxidant capacity, and nitrate and bioactive compounds content of beetroot gel and juice

	Beetroot gel	Beetroot juice
Nitrate^a^ (mmol 100 g^−1^)	6.3±0.41*	1.6±0.1
TAP (%)	87±0.1*	79.13±0.63
TPC^a^ (GAE mg·g^−1^)	1.98±0.03*	1.01±0.03
FRAP^a^ (µmol/ Fe^2+^·g^−1^)	16.115±50*	9.799±45.8
TEAC (ABTS % inhibition)	92.7±0.51*	86.7±0.66
Flavonids^a^ (QE mg·g^−1^)	1.37±0.03*	0.42±0.00
Saponins^a^ (mg·g^−1^)	22±0.54*	8.22±0.12

Values were expressed as mean±standard deviation (SD). The symbol * in same line indicates significant difference between samples (*p*<0.05; Bonferroni post-test).

a Values expressed at mL for beetroot juice.

The TPC concentration in the gel (1.98±0.03 mg g^−1^) was almost double the amount found in juice (1.01±0.03 mg g^−1^) (Table 3). Studies evaluated TPC in two commercial beet drinks used in clinical trials, observing values of 0.97 mg g^−1^ and 1.45 mg g^−1^, respectively (38), and in beets obtained by different forms of cultivation, where they found values of 0.51 mg g^−1^ in conventional beets and 0.6 mg g^−1^ in organic beets (39). In a recent study by Wruss et al. (33), TPC concentrations in juices from seven different beet varieties ranged from 0.88 to 1.29 mg g^−1^. The TPC values of beet gel are higher than those found for *in natura* beets and beet juice.

The flavonoid content of the beet gel was three times higher than the juice (0.37±0.03 vs. 0.42±0.00 QE mg g^−1^) (Table 3), indicating that the gel was enriched with these phenolic compounds. The effectiveness of flavonoid subclasses and flavonoid-rich food sources on risk factors for cardiovascular diseases are well documented (40).

TAC of the beetroot gel was evaluated by FRAP and TEAC assays, obtaining values of 16.12±0.05 µmol g^−1^ and 92.7±0.51%, respectively (Table 3). These values were significantly higher than those observed in the beet juice (9.8±0.04 µmol g^−1^ and 86.7±0.66%, respectively). Wootton-Beard and Ryan (38) evaluated the antioxidant capacity of 23 juices from several vegetables, including 2 beetroot juices, which presented FRAP values of 8,355 and 9,500 µmol g^−1^, which are half of the values of beetroot gel produced herein.

The total saponin content in the beetroot gel was of 22.00±0.54 mg g^−1^, 2.6 times higher than the value found in the beetroot juice processed in the laboratory (Table 3). Although few studies on the saponin content of beets are available, the levels of these compounds in the beetroot gel were also higher than those found by Mroczek (5), which showed saponin content ranging from 7.66 to 12.2 mg g^−1^ dry weight in three distinct beetroot cultivars (*Beta vulgaris* L.) Those authors found oleanolic acid as the main saponin in beetroot and this compound showed hypoglycemic effects.

### Microbiological quality

In Brazil, the RDC No. 12 of 2 January 2001 from the National Health Surveillance Agency (ANVISA) (41) established the sanitary microbiological standards of food for human consumption. The beetroot gel was evaluated according to the microbiological standards for fresh *in natura* prepared, dried, dehydrated, or freeze dried roots and tubers.

A value of 21 MPN g^−1^ of gel was found for total coliforms. Current law does not seek to limit contamination by total coliform, however, its presence in high counts indicates poor hygiene during product processing and storage. The coliform count in the present study was considered low compared to the count of 46 MPN g^−1^ found in sanitized beetroots stored at 5°C for 10 days (42).

No thermos tolerant (fecal) coliforms or *Salmonella* sp. were detected in the beetroot gel, which is a satisfactory result, because the maximum limit established by Brazilian legislation is of 10^3^ MPN g^−1^ for coliform and the absence of *Salmonella* sp.

The *B. cereus* count was of 3×10^2^ CFU g^−1^, below the current limit of 10^3^ CFU g^−1^. Taken together these results indicate that the beetroot gel was processed under satisfactory hygienic conditions as established by Brazilian legislation.

### Acceptance test

The beetroot gel with orange flavoring received higher mean scores in all sensory attributes (flavor, aroma, texture, and overall impression) than the gel with no flavoring (Table 4). The flavoring gel received average scores corresponding to ‘slightly like’ (score 6), indicating a product acceptance relation, while the unscented gel received average scores corresponding to ‘neither liked nor disliked’ (score 5), indicating an indifferent relationship of the product tasters. It is possible that orange flavoring positively influenced the appraisal of the texture and overall impression attributes by the evaluators.

**Table 4:** Sensory properties of beetroot gel samples evaluated by panelists

Attribute	Orange beetroot gel	Beetroot gel
Flavor	6.59±1.72*	5.48±1.96
Aroma	6.88±1.45*	5.42±1.75
Texture	6.85±1.56*	6.02±2
Overall acceptability	6.62±1.56*	5.7±1.66
Buying intention	3.42±1.21*	2.7±1.19

(1=dislike extremely, 9=like very much).

* Significant difference between samples (*p*<0.05; Tukey post-test).

The flavoring gel achieved a mean score of 3.4 in the purchase intent test, corresponding to ‘maybe would buy, maybe would not buy’, whereas the gel without the flavoring received a mean score of 2.7 corresponding to ‘possibly would not buy’, indicating rejection of this product.

The absence of information about the purpose of marketing the gel and its biological properties may have influenced the indifference observed in the evaluators purchase intent.

**Table 5:** Baseline characteristics of individuals

	Values
Subjects, *n*	5
Height, cm	1.72±0.07
Weight, Kg	69.4±9.8
Age, years	28±2
BMI, kg/m^2^	22.8±3
SBP, mm Hg	115±16.3
DBP, mm Hg	72±5.2
HR, bpm	69±9.5
Glucose, mg/dl	93±2.4
Cholesterol, mg/dl	168±37
LDL, mg/dl	50±19
HDL, mg/dl	46.8±6.2
Triglycerides, mg/dl	74.2±33
Urea (mg/dl)	27±5.9
Creatinine (mg/dl)	0.85±0.1
Uric acid (mg/dl)	3.15±1.4
TGO (U/L)	16.4±3.7
TGP (U/L)	20.5±6.3

Values are mean±SD. BMI, body mass index; SBP, systolic blood pressure; DBP, diastolic blood pressure; HR, heart rate; LDL, low density lipoprotein; HDL, high density lipoprotein; TGO, glutamic oxaloacetic transaminase; TGP, glutamic pyruvic transaminase.

### Plasma nitrite and BP evaluation after gel intake

**Fig. 2:**
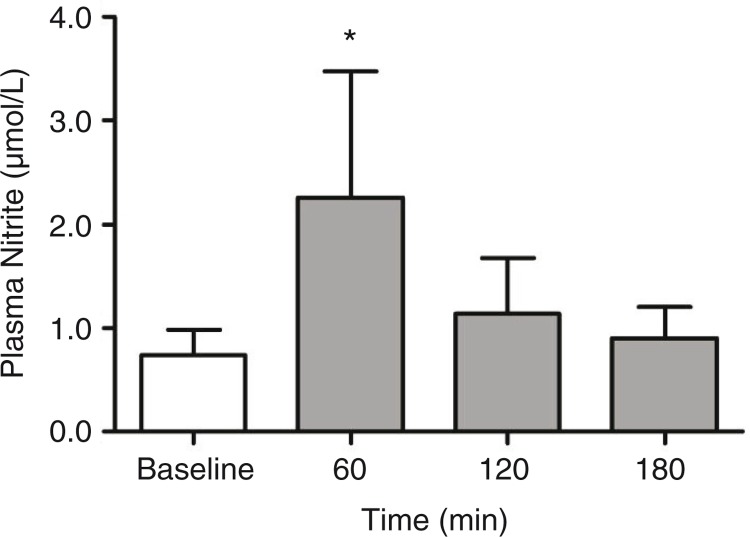
Nitrite plasma concentration in healthy subjects after beetroot gel supplementation. Plasma nitrite levels at baseline and after 60, 120, and 180 min of beetroot gel supplementation. The * symbol indicates significant difference in comparison to baseline (*p*<0.05; Dunnett's *post hoc* test). Values are expressed as mean±SD of triplicate determinations.

In a small-scale test with five healthy volunteers (Table 5), the beetroot gel showed no rejection, and no adverse effects were reported during the study. The mean nitrate concentration administered was 6.3 mmol 100 g^−1^, whereas nitrite concentrations showed a mean value of 0.003 mmol 100 g^−1^. A significant increase in plasma ${\text{NO}}_{\text{2}}^{-}$ after 60 min gel ingestion was observed, which returned to baseline after 180 min (Fig. 2). These results corroborate a previous study, which also observed a significant increase in ${\text{NO}}_{\text{2}}^{-}$ concentrations in plasma after the ingestion of 9.6 mmol ${\text{NO}}_{\text{3}}^{-}$ present in 140 mL of beetroot juice (26). However, in the present study, acute nitrate supplementation by the ingestion of beetroot gel increased plasma ${\text{NO}}_{\text{2}}^{-}$ threefold after 60 min, while in the previous, a 4-fold increase in plasma ${\text{NO}}_{\text{2}}^{-}$ was observed, but after 2.5 days of the beet juice ingestion.

**Fig. 3:**
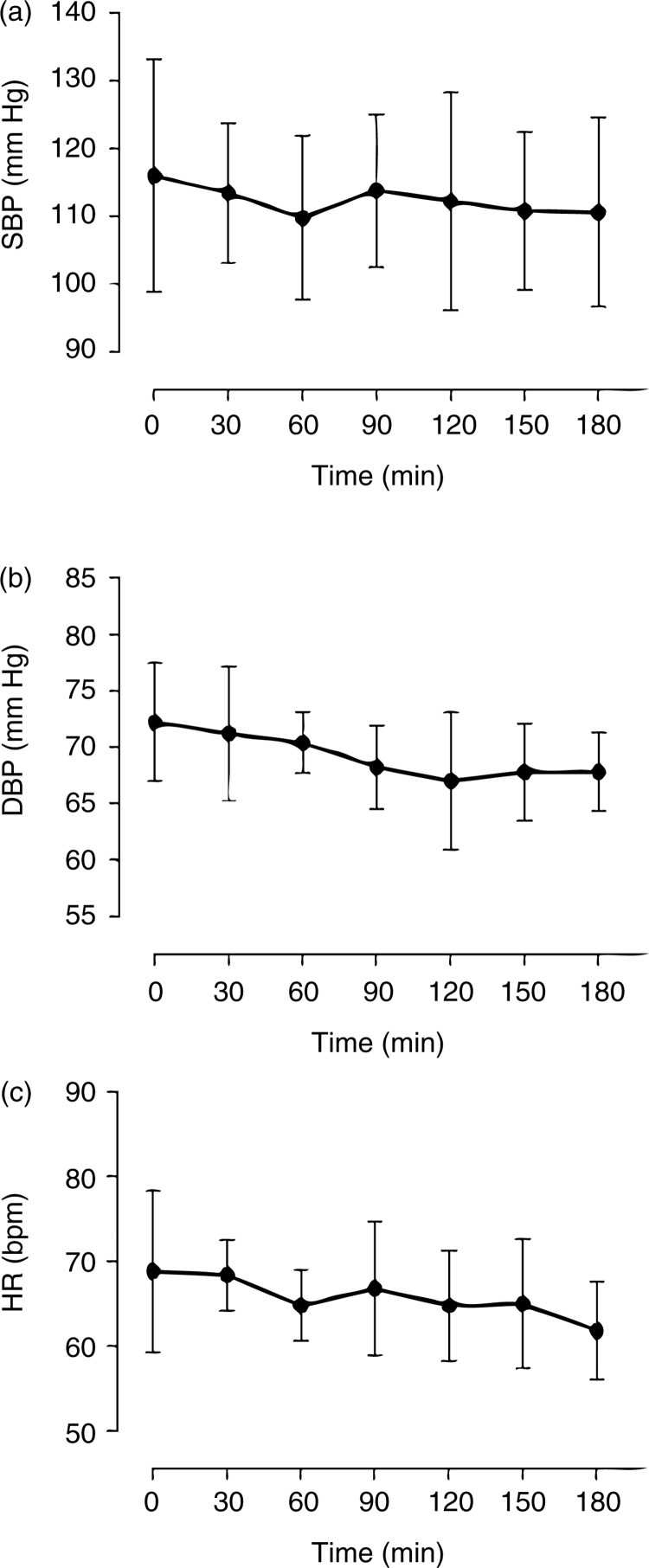
Blood pressure evaluation of healthy subjects. The effects of beetroot gel on SBP (a), DBP (b), and HR (c) were determined after acute nitrate supplementation by beetroot gel. Data are expressed as mean±SD from triplicate measurements taken from five healthy volunteers. The results not showed significant difference in comparison to baseline (*p*<0.05; Dunnett's *post hoc* test).

There was a mean SBP decrease of 6.2 mm Hg (5.3%) (Fig. 3), 60 min after the beetroot gel intake when compared to baseline levels. Likewise, a decrease of 5.2 mm Hg (7.2%) in the DPB 120 min after ingestion of the gel was also observed, as well as a decrease in HR (−7 bpm). Although these changes were statistically non-significant it must be considered that the study was performed with normotensives subjects. Miller et al. (9) observed a significant increase in plasma ${\text{NO}}_{\text{2}}^{-}$ after 3 days of beet juice intake (8.5 mmol ${\text{NO}}_{\text{3}}^{-}$) by eight normotensive subjects without significant changes on the SBP and DBP, similar to the results obtained herein.

There are several evidences regarding the contribution of dietary ${\text{NO}}_{\text{3}}^{-}$ from beetroot products in increasing NO production, via conversion to NO_2_(43, 44). According to Webb et al. (11), this bioactive ${\text{NO}}_{\text{2}}^{-}$ substantially lowers BP, inhibits platelet aggregation, and prevents endothelial dysfunctions in healthy individuals.

According to the guidelines of clinical management of primary hypertension in adults from the National Institute for Clinical Excellence (45), for each increase of 2 mmHg in SBP there is an increased risk of death from ischemic heart disease and cerebrovascular accident (stroke), whereas reductions from 5 to 7.5 mmHg in DBP were associated with decreases in the risks for stroke and coronary artery disease. These guidelines emphasize the importance of the possible effect promoted by the gel on BP if ingested by individuals with risk factors for cardiovascular disease.

## Conclusions

The nitrate-enriched beetroot gel presented higher fiber, carbohydrate, and antioxidant content than beet juice, associated with advantageous rheological properties for oral administration and handling in the nutritional supplement industry. The nitrate content of the beetroot gel caused an increase in ${\text{NO}}_{\text{2}}^{-}$ plasma levels and a tendency to lower BP in healthy subjects. The results shown herein encourage the testing of beetroot gel in larger groups of healthy, physically active subjects, as well as in pre-hypertensive and dyslipidemic individuals.

## References

[1] Lange W, Brandenburg WA, De bock TS (1999). Taxonomy and cultonomy of beet (*Beta vulgaris* L.). Bot J Linn Soc.

[2] United States Department of Agriculture (2013). USDA national nutrient database for standard reference.

[3] Rink SM, Mendola P, Mumford SL, Poudrier KJ, Browne RW, Wactawski-Wende J (2013). Self-report of fruit and vegetable intake that meets the 5 a day recommendation is associated with reduced levels of oxidative stress biomarkers and increased levels of antioxidant defense in premenopausal women. J Acad Nutr Diet.

[4] Machha A, Schechter AN (2011). Dietary nitrite and nitrate: a review of potential mechanisms of cardiovascular benefits. Eur J Nutr.

[5] Mroczek A, Kapusta I, Janda B, Janiszowska W (2012). Triterpene saponin content in the roots of red beet (*Beta vulgaris* L.) cultivars. J Agric Food Chem.

[6] Georgiev VG, Weber J, Kneschke EM, Denev PN, Bley T, Pavlov AI (2010). Antioxidant activity and phenolic content of betalain extracts from intact plants and hairy root cultures of the red beetroot *Beta vulgaris* cv. Detroit dark red. Plant Foods Hum Nutr.

[7] Coles LT, Clifton PM (2012). Effect of beetroot juice on lowering blood pressure in free-living, disease-free adults: a randomized, placebo-controlled trial. Nutr J.

[8] Lundberg JO, Weitzber E (2005). NO generation from nitrite and its role in vascular control. Arterioscler Thromb Vasc Biol.

[9] Miller GD, Marsh AP, Dove RW, Beavers D, Presley T, Helms C (2012). Plasma nitrate and nitrite are increased by a high-nitrate supplement but not by high-nitrate foods in older adults. Nutr Res.

[10] Kapil V, Khambata RS, Robertson A, Caulfield MJ, Ahluwalia A (2014). Dietary nitrate provides sustained blood pressure lowering in hypertensive patients: a randomized, phase 2, double-blind, placebo-controlled study. Hypertension.

[11] Webb AJ, Patel N, Loukogeorgakis S, Okorie M, Aboud Z, Misra S (2008). Acute blood pressure lowering, vasoprotective, and antiplatelet properties of dietary nitrate via bioconversion to nitrite. Hypertension.

[12] Lundberg JO, Gladwin MT, Ahluwalia A, Benjamin N, Bryan NS, Butler A (2009). Nitrate and nitrite in biology, nutrition and therapeutics. Nat Chem Biol.

[13] Breese BC, McNarry MA, Marwood S, Blackwell JR, Bailey SJ, Jones AM (2013). Beetroot juice supplementation speeds O_2_ uptake kinetics and improves exercise tolerance during severe-intensity exercise initiated from an elevated metabolic rate. Am J Physiol Regul Integr Comp Physiol.

[14] AOAC (2012). Official methods of analysis.

[15] Li H, Meininger CJ, Wu G (2000). Rapid determination of nitrite by reversed phase high-performance liquid chromatography with fluorescence detection. J Chromatogr B Analyt Technol Biomed Life Sci.

[16] Glód BK, Piszcz P, Czajka K, Zarzycki PK (2011). A new total antioxidant potential measurements using RP-HPLC assay with fluorescence detection. J Chromatogr Sci.

[17] Singleton VL, Orthofer R, Lamuela-Raventós RM (1999). Analysis of total phenols and other oxidation substrates and antioxidants by means of folin-ciocalteu reagent. Oxid Antioxid Part A.

[18] Taie HAA, El-Mergawi R, Radwan S (2008). Isoflavonoids, flavonoids, phenolic acids profiles and antioxidant activity of soybean seeds as affected by organic and bioorganic fertilization. Am Eurasian J Agric Environ Sci.

[19] Shiau I, Shih T, Wang Y, Chen H (2009). Quantification for saponin from a soapberry (*Sapindus mukorossi* Gaertn) in cleaning products by a chromatographic and two colorimetric assays. J Fac Agric Kyushu U.

[20] Benzie IF, Strain JJ (1996). The ferric reducing ability of plasma (FRAP) as a measure of “antioxidant power”: the FRAP assay. Anal Biochem.

[21] Re R, Pellegrini N, Proteggente A, Pannala A, Yang M, Rice-Evans C (1999). Antioxidant activity applying an improved ABTS radical cation decolorization assay. Free Radic Biol Med.

[22] American Public Health Association (2005). Standard methods for the examination of water and wastewater.

[23] Drake MA (2007). Invited review: sensory analysis of dairy foods. J Dairy Sci.

[24] Meilgaard MC, Carr T, Civille GV (2007). Sensory evaluation techniques.

[25] Griesenbeck JS, Steck MD, Huber JC, Sharkey JR, Rene AA, Brender JD (2009). Development of estimates of dietary nitrates, nitrites, and nitrosamines for use with the Short Willet Food Frequency Questionnaire. Nutr J.

[26] Kelly J, Fulford J, Vanhatalo A, Blackwell JR, French O, Bailey SJ (2013). Effects of short-term dietary nitrate supplementation on blood pressure, O_2_ uptake kinetics, and muscle and cognitive function in older adults. Am J Physiol Regul Integr Comp Physiol.

[27] Rao MA (2014). Rheology of fluid, semisolid and solid foods: principles and applications.

[28] Ravichandran K, Saw NMMT, Mohdaly AAA, Gabr MMA, Kastell A, Riedel H (2013). Impact of processing of red beet on betalain content and antioxidant activity. Food Res Int.

[29] Gengatharan A, Dykes GA, Choo WS (2015). Betalains: natural plant pigments with potential application in functional foods. LWT – Food Sci Technol.

[30] Jeukendrup AA (2014). Step towards personalized sports nutrition: carbohydrate intake during exercise. Sports Med.

[31] Rodriguez NR, Di Marco NM, Langley S, American College of Sports Medicine (ACSM) (2009). Nutrition and athletic performance. Med Exerc.

[32] Threapleton DE, Greenwood DC, Evans CE, Cleghorn CL, Nykjaer C, Woodhead C (2013). Dietary fibre intake and risk of cardiovascular disease: systematic review and meta-analysis. Br Med J.

[33] Wruss J, Waldenberger G, Huemer S, Uygun P, Lanzerstorfer P, Müller U (2015). Compositional characteristics of commercial beetroot products and beetroot juice prepared from seven beetroot varieties grown in Upper Austria. J Food Compost Anal.

[34] Weitzberg E, Lundberg JO (2013). Novel aspects of dietary nitrate and human health. Annu Rev Nutr.

[35] Kapil V, Milsom AB, Okorie M, Maleki-Toyserkani S, Akram F, Rehman F (2010). Inorganic nitrate supplementation lowers blood pressure in humans: role for nitrite-derived NO. Hypertension.

[36] Joris PJ, Mensink RP (2013). Beetroot juice improves in overweight and slightly obese men postprandial endothelial function after consumption of a mixed meal. Atherosclerosis.

[37] Glod BK, Piszcz P, Czajka J, Zarzycki PK (2012). Evaluation of total antioxidant potential of selected biogenic polyamines, non-alcoholic drinks and alcoholic beverages using improved RP-HPLC assay involving fluorescence detection. Food Chem.

[38] Wootton-Beard PC, Moran A, Ryan L (2011). Stability of the antioxidant capacity and total polyphenol content of 23 commercially available vegetable juices before and after in vitro digestion as measured by FRAP, DPPH, ABTS and Folin Ciocalteu methods. Food Res Int.

[39] Bavec M, Turinek M, Grobelnik-Mlakar S, Slatnar A, Bavec F (2010). Influence of industrial and alternative farming systems on contents of sugars, organic acids, total phenolic content, and the antioxidant activity of red beet (*Beta vulgaris* L. ssp. vulgaris Rote Kugel). J Agric Food Chem.

[40] Hooper L, Kroon PA, Rimm EB, Cohn JS, Harvey I, Le Cornu KA (2008). Flavonoids, flavonoid-rich foods, and cardiovascular risk: a meta-analysis of randomized controlled trials. Am J Clin Nutr.

[41] Brazill. National Health Surveillance Agency (2001) Technical Regulation on microbiological standards for foods.

[42] Vitti MCD, Kluge RA, Gallo CR, Schiavinato MA, Moretti CL, Jacomino AP (2004). Physiological and microbiological aspects of fresh cut beet roots. Pesqui Agropecu Bras.

[43] Siervo M, Lara J, Ogbonmwan I, Mathers JC (2013). Inorganic nitrate and beetroot juice supplementation reduces blood pressure in adults: a systematic review and meta-analysis. J Nutr.

[44] Lara J, Ashor AW, Oggioni C, Ahluwalia A, Mathers JC, Siervo M (2015). Effects of inorganic nitrate and beetroot supplementation on endothelial function: a systematic review and meta-analysis. Eur J Nutr.

[45] National Clinical Guideline Center (2011). NICE Clinical Guidelines 127. Hypertension: The clinical management of primary hypertension in adults.

